# Evidence for rapid adaptive evolution of tolerance to chemical treatments in *Phytophthora* species and its practical implications

**DOI:** 10.1371/journal.pone.0208961

**Published:** 2018-12-10

**Authors:** Shannon Hunter, Nari Williams, Rebecca McDougal, Peter Scott, Matteo Garbelotto

**Affiliations:** 1 Department of Biology, School of Science, University of Waikato, Hamilton, New Zealand; 2 Department of Environmental Science, Policy & Management, University of California, Berkeley, CA, United States of America; 3 Forest Protection, Scion, Rotorua, New Zealand; Agriculture and Agri-Food Canada, CANADA

## Abstract

Chemical treatments are used widely in agricultural and natural settings to protect plants from diseases; however, they may exert an important selection pressure on plant pathogens, promoting the development of tolerant isolates through adaptive evolution. Phosphite is used to manage diseases caused by *Phytophthora* species which include a large number of the most economically damaging plant pathogens worldwide. Phosphite controls the growth of *Phytophthora* species *in planta* without killing it; as a result, isolates can develop tolerance to phosphite after prolonged exposure. We investigated the inter- and intra-specific variability in phosphite tolerance of eleven *Phytophthora* species, including *P*. *ramorum*, an internationally important, highly regulated pathogen. *Phytophthora ramorum is* a good model system because it is comprised of multiple genetically homogeneous lineages. Seven species were found to be consistently sensitive to phosphite based on the low Effective Concentration (EC) 50 values of all isolates tested (amount of phosphite required to inhibit mycelial growth by 50% relative to growth in the absence of phosphite). However, *P*. *ramorum*, *P*. *lateralis*, *P*. *crassamura* and *P*. *cambivora* showed intraspecific variability in sensitivity to phosphite, with at least one isolate showing significantly higher tolerance than the other isolates. Within the three *P*. *ramorum* evolutionarily divergent lineages tested, NA1 was the most susceptible to phosphite, the NA1 and EU1 lineages showed intralineage variability and the NA2 lineage showed a decreased sensitivity to phosphite overall as all isolates were relatively tolerant. This finding is relevant because NA1 is dominant in the wild and can be controlled using phosphite, while the EU1 lineage has recently been identified in the wild and is phosphite-tolerant, making the treatment approach potentially less effective. *Phytophthora ramorum*, *P*. *lateralis* and *P*. *crassamura* are either selfing, homothallic species, or are known to reproduce exclusively clonally, indicating tolerance to phosphite can emerge even in the absence of sexual recombination.

## Introduction

Adaptive evolution by invasive plant pathogens, including the development of tolerance to chemical treatments, presents an ongoing threat to plant health, influencing agricultural sustainability and food security [1]. Adaptive evolution is the process by which an organism changes in response to exposure to novel selection pressures. Tolerance to chemical treatments is an important adaptive trait of plant pathogens, and chemical resistance is a concern for nursery, horticulture, and plant production industries, as well as for the health of native ecosystems. The development of resistance to chemicals (e.g. chemicals present in nature) can occur on an evolutionary time scale and is correlated to: a) the intensity and duration of exposure, b) size of the population exposed, c) its generation time, and, d) the innate constitutive ability of a species to survive in presence of genomic structural variation associated with differential gene expression. When they are the target of chemical treatments, some pathogen species may progressively evolve tolerance through evolutionary processes of varying lengths. Other species may not develop tolerance either because their genome is not prone to survive genomic re-assortments, or because they have not been under selection pressure. If selection pressure is constant then the tolerance trait may become fixed in a species and be a common phenotype of all individuals [2].

Variation within a species is instead the trademark of a recent and much faster process. If the species in question is genetically homogeneous and panmictic, then we can assume that differences in tolerance may have been triggered by differential exposure of different individuals to the selection pressure. These cases are important because a) they may help us understand how tolerance evolves, b) they uncover diversity in history within a pathosystem, and c) they may highlight a process that greatly enhances the ability of exotic pathogens to adapt to novel environments and may explain the invasive potential of a species. The speed at which an organism can adapt is pivotal especially for emergent invasive species. Sexual recombination and hybridization with associated gene introgression and changes in ploidy may lead to rapid differential gene expression that in turn may help to develop resistance. Rapid adaptation without sex and recombination is unexpected and potentially a much more intriguing process.

We used a selection of *Phytophthora* species to study rapid adaptive evolution. *Phytophthora* species cause many major plant disease epidemics worldwide, impacting horticulture, plant trade and production planting industries through the loss of plants and the costs arising from disease management. We identified exposure to phosphite as a selection pressure of relevance because phosphite may be the only active ingredient used in plant production facilities, as well as in agricultural and natural settings [3]. Isolates of *P*. *cinnamomi* have been shown to be tolerant to phosphite after prolonged exposure in avocado orchards both in Australia [4] and South Africa [5, 6]. We chose the genus *Phytophthora*, because it is known that species within this genus differ significantly in the amount of intraspecific genetic diversity, and in the way novel diversity is generated. We included isolates of the *P*. *ramorum* lineages, known to cause disease in natural settings in European countries (EU1 lineage) and in the Western United states (NA1 and recently EU1 lineages) [7]. Some *Phytophthora* species follow the well-known process through which mutation and sexual recombination operate together to generate genetic and underlying phenotypic diversity. However, some species reproduce asexually resulting in widespread and apparently identical clonal genotypes, and in novel genotypes generated exclusively through mutation [8] or through genomic reassortments [9, 10]. The presence of clonal and putative identical genotypes is an ideal set up to evaluate the potential of a species to adapt and to understand the basis of this evolutionary process without the confounding factor of significant genetic variation among individuals.

Based on the literature, tolerance to chemicals in microbes can evolve due to the insurgence of recombined alleles through mating [11] or by different expression of extant genes due to changes in either ploidy or copy number of genes through genomic duplication [12]. Additional examples include resistance to Quinone outside-inhibiting (Qol) fungicides in populations of *Plasmopara viticola*, the causal agent of downy mildew, based on a mutation in the cytochrome b gene [13]; and flumorph resistance in *Phytophthora capsici* regulated by multiple mechanisms involving but not limited to a decrease in the biosynthesis of amino acids and a shift in energy generation [14].

The aims of this study were to:

1. identify *in vitro* the overall difference in phosphite tolerance among 11 *Phytophthora* species to create a broad frame of reference on the abundance of the trait within a select but significant group of species,
2. identify variation within species as an example of differential responses linked to different history of phosphite exposure, and
3. identify those cases of putative rapid evolution e.g. cases where tolerance varies within clonal or quasi clonal individuals in a taxonomic entity characterized by low genetic diversity.

## Methods

### Experimental design

This study examined the phosphite sensitivity of 77 *Phytophthora* isolates from 11 species maintained at the University of California, Berkeley collection (Table 1). All species tested have been detected in the wild [15]. Sensitivity to phosphite was tested at six concentrations, including 0, 15, 40, 80, 200 and 500 μg/mL.

**Table 1:** *Phytophthora* isolates used in this study.

Species	ID	Host	Substrate	Location	Location type	Date collected	Mating type/ Lineage/ Haplotype
*Phytophthora cryptogea*	MBP-B-DIAU1.1	*Diplacus aurantiacus*	root bait	Marin, California, USA	Restoration site		
	CCWN-295B-DIAU5.1	*D*. *aurantiacus*	root bait	Santa Cruz, California, USA	Nursery		
* *	MBP-B-DIAU4.1	*D*. *aurantiacus*	root bait	Marin, California, USA	Restoration site		
* *	CCW-DP-DIAU-ROOTS.1	*D*. *aurantiacus*	root isolation	Santa Cruz, California, USA	Nursery		
* *	NPC-79B-MIAU.1	*D*. *aurantiacus*	root bait	San Francisco, California, USA	Restoration site		
* *	ENPN122-DP-DIAU12.1	*D*. *aurantiacus*	root isolation	Santa Cruz, California, USA	Nursery		
* *	ENPN123-DP-DIAU13.2	*D*. *aurantiacus*	root isolation	Santa Cruz, California, USA	Nursery		
* *	ENPN80-B-DIAU10.2	*D*. *aurantiacus*	root bait	Santa Cruz, California, USA	Nursery		
*P*. *multivora*	FOFU-C2-CETH.1	*Ceanothus thrysiflorus*	root bait	San Francisco, California, USA	Nursery		
* *	NPC-47B-CETH.1	*C*. *thrysiflorus*	root bait	San Francisco, California, USA	Restoration site		
* *	MA-33B-FRCA.1	*Frangula californica*	root bait	Marin, California, USA	Restoration site		
* *	MA-60B-FRCA.1	*F*. *californica*	root bait	Marin, California, USA	Restoration site		
*P*. *crassamura*	PLRA-SFPUC.1	*Platanus racemosa*	bark canker	Alameda, California, USA	Restoration site		i
* *	PLRA-DRYSOIL1A.1	*P*. *racemosa*	root bait	Alameda, California, USA	Restoration site		i
* *	TEVA-59B-ALRU.1	*Alnus rubra*	root bait	Marin, California, USA	Nursery		i
* *	TEVA-326B-JUEF.1	*Juncus effusus*	root bait	Marin, California, USA	Nursery		i
* *	SM-45B-FRCA.1	*F*. *californica*	root bait	San Mateo, California, USA	Restoration site		iib
* *	SM-39B-FRCA.1	*F*. *californica*	root bait	San Mateo, California, USA	Restoration site		iib
* *	FOR-OUT-06B.1	* *	root bait	San Francisco, California, USA	Nursery		iib
* *	MA-85B-SOIL.1		root bait	Marin, California, USA	trail stock area		iib
*P*. *megasperma*	MBP-DIAU10-DPSTEM.1	*D*. *aurantiacus*	bark canker	Marin, California, USA	Restoration site		
* *	MBP-DIAU5-DPSTEM.1	*D*. *aurantiacus*	bark canker	Marin, California, USA	Restoration site		
* *	MBP-DIAU4-DPSTEM.1	*D*. *aurantiacus*	bark canker	Marin, California, USA	Restoration site		
* *	MBP-B-DIAU10.1	*D*. *aurantiacus*	root bait	Marin, California, USA	Restoration site		
*P*. *cactorum*	7-HR.1	*Heteromeles arbutifolia*	root bait	Orange, California, USA	Nursery		
* *	7-HP.1	*H*. *arbutifolia*	root bait	Orange, California, USA	Nursery		
* *	PNPN-C-39FRCA.1	*F*. *californica*	root bait	San Francisco, California, USA	Nursery		
* *	10-SP.1	*Salix lasiolepis*	root bait	Orange, California, USA	Nursery		
* *	AKWA	* *		Alaska			
* *	7912.1	* *					
* *	117R	*Alnus rubra*	root isolation	Lincoln, Oregon	Riparian zone		
* *	MP19	*Pseudotsuga menziesii*		Oregon			
*P*. *cambivora*	MP21	Almond		Chico, CA		1980	
* *	MP28	Apple		Ulster, NY		1983	A1
* *	L.170.B.HEAR	* *					
* *	FOR.61B.HEAR	* *					
* *	NPL.22B.HEAR	* *					
*P*. *cinnamomi*	MC11	White Fir		Eldorado, CA	Nursery		A2
* *	P.2021 COFFEY	*Camelia japonica*		California			A1
* *	P.3662 COFFEY	*Araucaria* sp.		Papua New Guinea			A2
* *	P.6377 COFFEY	* *	Soil	Taiwan			A2
* *	P.6493 COFFEY	*Rhodendron* sp.		China			A1
*P*. *nemorosa*	P.106	*Umbellularia californica*		Marin, California, USA	Samuel P. Taylor State park		
* *	P.113	*U*. *californica*		Marin, California, USA	Samuel P. Taylor State park		
* *	P.114	*U*. *californica*		Marin, California, USA	Samuel P. Taylor State park		
* *	P.115	*U*. *californica*		Marin, California, USA	Samuel P. Taylor State park		
* *	1050 Hansen1	*Lithocarpus densiflora*		Oregon		30/07/1998	
* *	2052.2 Hansen2	*L*. *densiflora*		Oregon		9/10/1997	
* *	2059.4 Hansen6	*L*. *densiflora*		Oregon		17/10/1997	
* *	5104 Hansen22	*Myrtle*	Myrtle leaf	Oregon		9/12/1997	
*P*. *lateralis*	PL-9	*Chamaecyparis lawsoniana*		Baker Flat, Del Norte, CA		25/08/2004	
* *	PL-25	*Taxus brevifolia*		Middle forth Smith, Del Norte, CA		25/08/2004	
* *	Pl-28	* *		Lawson Creek, Del Norte, CA		25/08/2004	
* *	PL-31	* *	Soil	Del Norte, CA		25/08/2004	
* *	PL-34	*Taxus brevifolia*		Panther Ridge, Del Norte, CA		25/08/2004	
* *	PL-47	*T*. *brevifolia*		Monkey Creek, Del Norte, CA		25/08/2004	
* *	PL-54	*T*. *brevifolia*		Gordon Creek, Del Norte, CA		25/08/2004	
*P*. *syringae*	MP-12	*Rhododendron* sp.		Santa Cruz, CA	Nursery, Scotts Valley	1/04/2002	
* *	MP-15	*Rhododendron* sp.		Santa Cruz, CA	Nursery, Scotts Valley	1/04/2002	
	KDA_RT9						
	SM15FEB_5CRP						
	SM15APR_B0V						
	SM15FEB_HOP						
	BSP2014_502						
*P*. *ramorum*	SI-556	* *	stream monitoring			2012	EU1
* *	SI-592	* *	stream monitoring			2012	EU1
* *	SI-595	* *	stream monitoring			2012	EU1
* *	MR-59	Rhododendron cv. Colonel Coen	Isolated from asymptomatic plants	Sacramento, Co	Nursery	2005	NA2
* *	MR-64	Rhododendron cv. Colonel	Isolated from asymptomatic roots	Sacramento, Co	Nursery	2005	NA2
* *	MR-69	Rhododendron cv. Colonel	Isolated from asymptomatic plants	Sacramento, Co	Nursery	2005	NA2
* *	MR-88	Rhododendron cv. Colonel	Isolated from asymptomatic plants	Sacramento, Co	Nursery	2005	NA2
* *	MR-126	*U*. *californica*		Marin, CA	Angel island	2005	NA1
* *	MR-180	*U*. *californica*		Marin, CA	Devil's Glich (Samuel P.Taylor)	2005	NA1
* *	MR-268	* *		Humboldt, CA		2005	NA1
* *	MR-270	* *		Humboldt, CA		2005	NA1
* *	MR-187	*U*. *californica*		Monterey, CA	Big Sur/Deetjen's Inn	2005	NA1
* *	MR-196	*U*. *californica*		Monterey, CA	Big Sur/Deetjen's Inn	2005	NA1
* *	1461	*U*. *californica*		San Mateo, CA		2005	NA1

### *Phytophthora* isolates

*Phytophthora* isolates from the University of California, Berkeley culture collection were maintained in water vials at 4°C on 10% V8 agar containing 100 mL/L Campbell’s V8 juice, 900 mL/L deionised water, 15g/L agar and 1 g/L CaCO_3_. Isolates were sub-cultured onto 10% V8 agar five days prior to the experiment.

### Phosphite amendment

The phosphite used was Agri-Fos® Systemic Fungicide (Agrichem, Yatala QLD, Australia), a commercial potassium phosphite fungicide containing 400 g/L phosphorous acid, present as mono- and di-potassium phosphonate. A stock solution containing 100 g/L of phosphite was filter sterilised using 0.22 μm pore filters (VWR International, Radnor, Pennsylvania, USA) then added to autoclaved media when it had cooled to approximately 50°C.

### Phosphite medium

Growth experiments were conducted in 90 mm Petri dishes (Thermo Fisher, San Francisco, California, United States) containing 20 mL of V8 10% agar amended with phosphite. The inoculum consisted of a 5 mm mycelial plug from the growing margin of a five-day old colony on 10% V8. Two perpendicular measurements of colony diameter were taken 5 days after inoculation, the diameter of the inocuum plug was subtracted to get the diameter of the mycelium. A second measurement of the *P*. *lateralis* isolates was taken 7 days after inoculation due to the slow growth rate of this species. The day 7-measurement of *P*. *lateralis* was used for the analysis.

### Data analysis

Percentage growth of the isolates at each of the phosphite concentrations was calculated as a percentage of the growth in the absence of phosphite. The EC50 was calculated by plotting percentage inhibition against log_10_ phosphite concentration and using the equation from a logarithmic trendline [16, 17]. Data analysis was done using the program R version 3.2.4 [18].

We used K means clustering for EC50 values for each of the isolates to determine whether isolates of the various *Phytophthora* species employed in this study were equally susceptible or whether intraspecific variation existed among isolates. K-means clustering is a variation on conventional cluster analysis, in which the investigators designate the number of groups into which the cases (in this study, the *Phytophthora* isolates) will be clustered [19]. Using the EC50 values, we specified two clusters, seeking to force a classification into two groups. In this case, the results are useful if isolates from within a species cluster into both groups. If any species clusters only into one group then it can be considered sensitive or tolerant in comparison to the other isolates tested.

## Results

### Phosphite sensitivity

**Fig 1:**
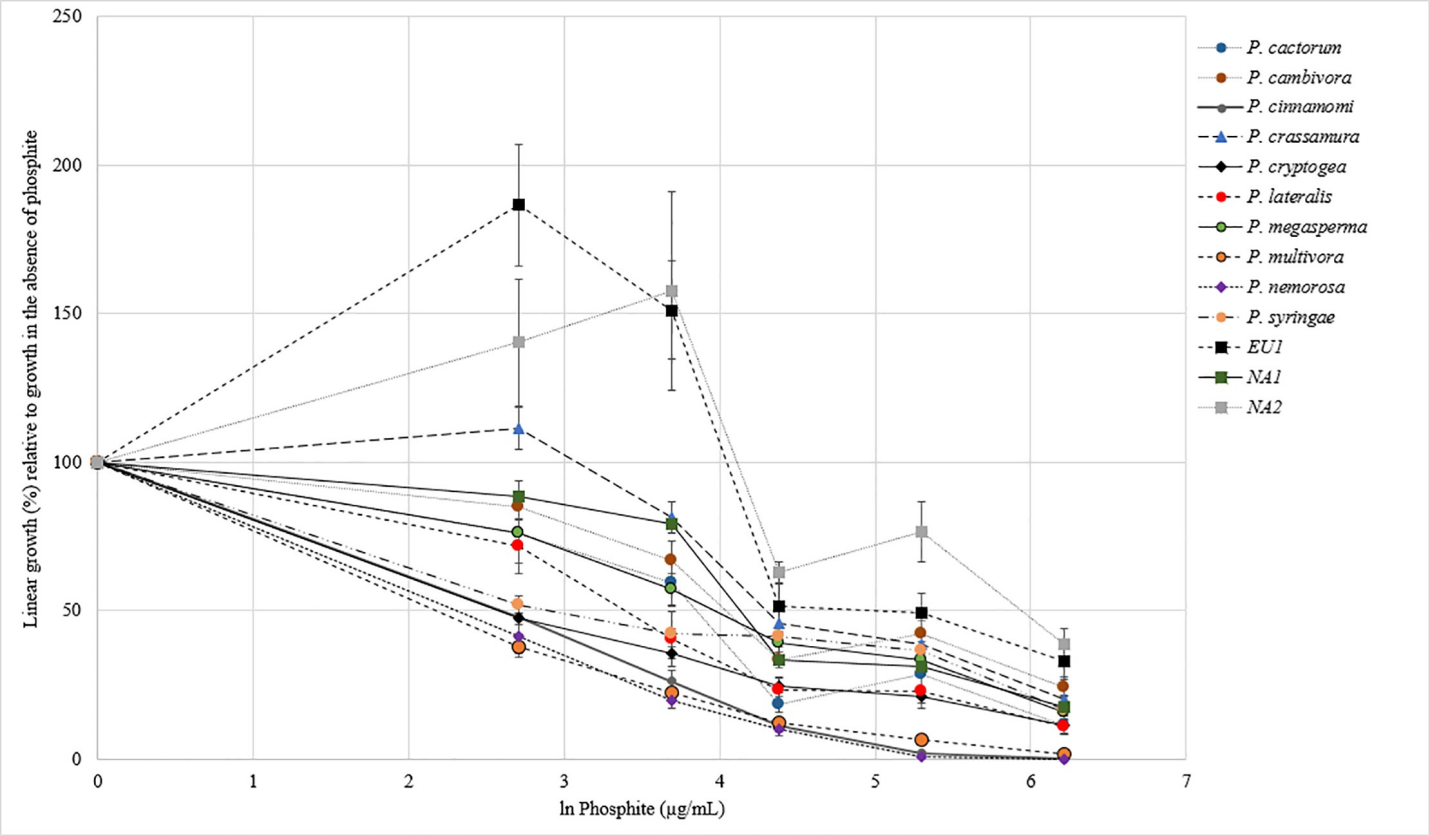
Average growth curves of the 11 *Phytophthora* species. Growth is expressed as percentage growth relative to growth in the absence of phosphite. Error bars are standard error. *P*. *ramorum* is split into the three lineages EU1, NA1 and NA2. The x axis is the natural log of phosphite concentration of the treatments 0, 15, 40, 80, 200 and 500 μg/mL phosphite.

Interspecific variability in tolerance to phosphite was observed among the *Phytophthora* species tested, with *P*. *cinnamomi*, *P*. *nemorosa* and *P*. *multivora* showing higher sensitivity to phosphite and *P*. *ramorum* lineages NA2 and EU1 showing lower sensitivity to phosphite (Fig 1).

On average, growth was promoted on the two lowest phosphite concentrations (relative to growth on the control) for the *P*. *ramorum* lineages NA2 and NA1 (Fig 1). Growth was also promoted for *P*. *crassamura* on the 15 μg/mL phosphite treatment relative to the control (Fig 1).

**Fig 2:**
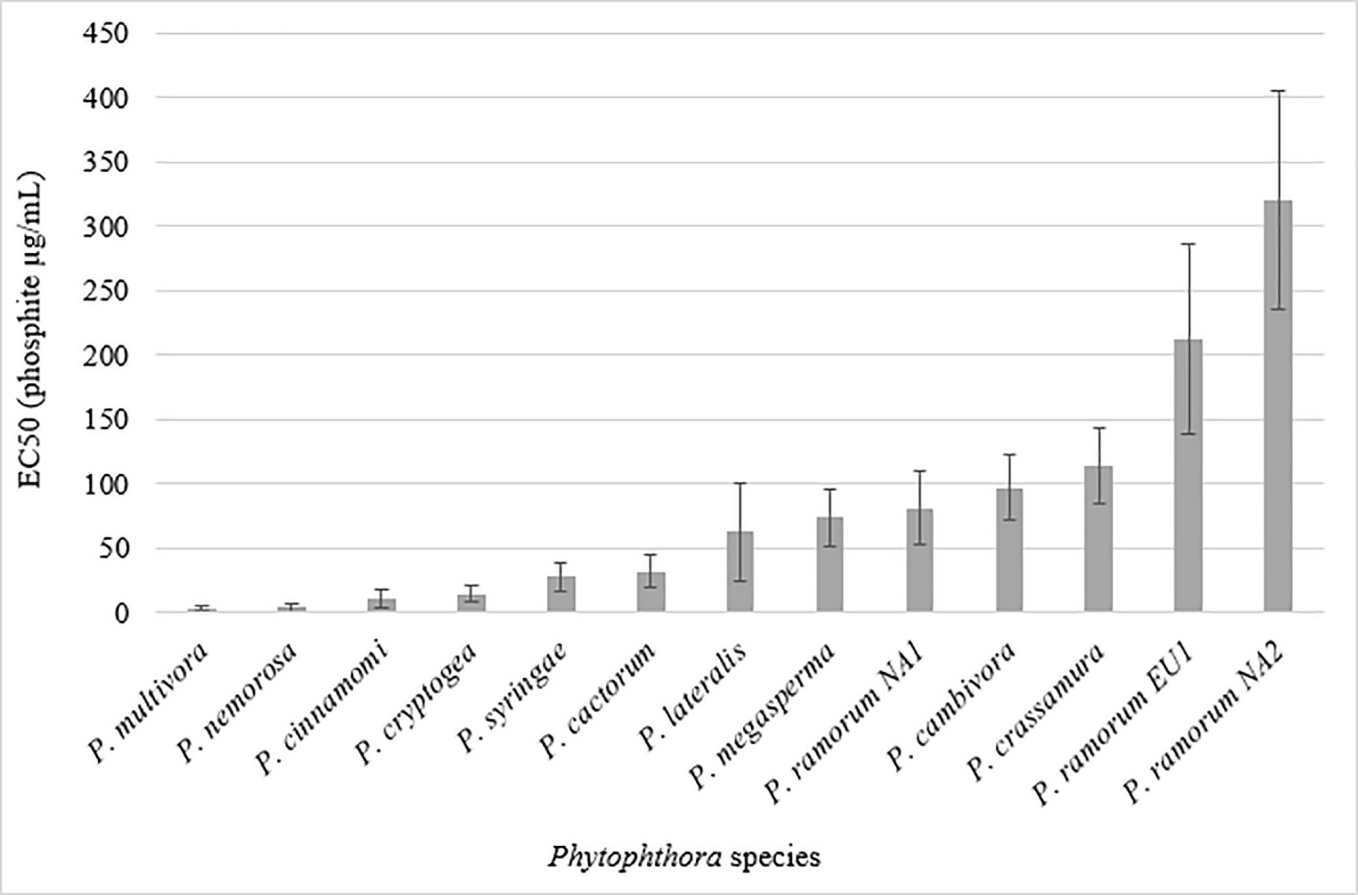
Average EC50 values of the *Phytophthora* species. The *P*. *ramorum* lineages NA1, NA2 and EU1 are shown separately. Error bars are standard error.

*Phytophthora multivora*, *P*. *nemorosa* and *P*. *cinnamomi* were effectively unable to grow on the two highest phosphite concentrations (200 and 500 μg/mL phosphite) and they had the lowest EC50 values on average (Fig 2).

The average EC50 value for the *P*. *ramorum* lineage NA2 was significantly higher than all other species (except the lineage EU1) (Fig 2).

**Fig 3:**
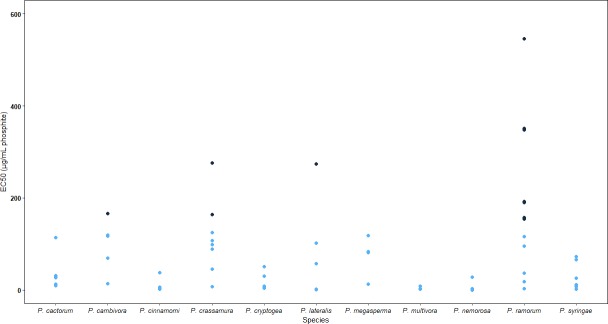
K means analysis showing phosphite sensitive (blue dots) and more phosphite tolerant isolates (black dots). The sensitive isolates (blue dots) had a mean EC50 of 33.8 **μ**g/mL phosphite and the more tolerant isolates (black dots) had a mean EC50 of 251.0 **μ**g/mL phosphite. Isolates from *P*. *cambivora*, *P*. *crassamura*, *P*. *lateralis* and *P*. *ramorum* clustered in both groups, the isolates of the remaining species all clustered in the more sensitive group 1.

Intraspecific variability in phosphite tolerance was observed for *P*. *ramorum*, *P*. *crassamura*, *P*. *lateralis* and *P*. *cambivora* as isolates of these species clustered in both the phosphite sensitive (mean EC50 of 251.0 μg/mL phosphite) and tolerant (mean EC50 of 33.8 μg/mL phosphite) groups of a K means analysis (Fig 3).

All the isolates of *P*. *cactorum*, *P*. *cinnamomi*, *P*. *cryptogea*, *P*. *multivora*, *P*. *megasperma*, *P*. *nemorosa*, and *P*. *syringae* were grouped in the more sensitive cluster (Fig 3). Intraspecific variation in phosphite tolerance was clear in the *P*. *ramorum* lineages NA1 and EU1 as the isolates were spread across both groups while the NA2 isolates all clustered in the tolerant group (Table 2).

**Table 2:** EC50 values and clustering of the *Phytophthora ramorum* isolates (sorted by lineage) in the phosphite-sensitive group 1 (mean EC50 value of 33.8 μg/mL phosphite) and in the phosphite-tolerant group 2 (mean EC50 value of 251.0 μg/mL phosphite) based on the K means analysis.

Lineage	Isolate	EC50	Cluster
NA1	MR-187	2.6	1
	MR-126	18.1	1
	MR-268	36.9	1
	1461	116.5	1
	MR-196	154.2	2
	MR-270	158.0	2
EU1	SI-595	95.5	1
	SI-556	192.0	2
	SI-592	348.4	2
NA2	MR-59	190.4	2
	MR-88	191.7	2
	MR-69	351.6	2
	MR-64	546.3	2

There was a significant difference between the EC50 values of the NA1 isolates grouped in cluster 1 and 2 (two sample t-test assuming unequal variances p = 0.02). The NA2 isolates were more tolerant than the NA1 on average (two sample t-test assuming unequal variances p = 0.055).

Intraspecific variation in phosphite tolerance was also observed in *P*. *cambivora*, *P*. *crassamura* and *P*. *lateralis* (Table 3).

**Table 3:** One isolate of each *Phytophthora cambivora* and *P*. *lateralis* and two of *P*. *crassamura* clustered in the phosphite tolerant group 2 (mean EC50 of 251.0 μg/mL phosphite) and the remaining isolates clustered in the more phosphite sensitive group 1 (mean EC50 of 33.8 μg/mL phosphite) in the K means analysis.

Species	Isolate	Mating type/ Haplotype	EC50	Cluster
*P*. *cambivora*	MP21		13.6	1
* *	FOR.61B.HEAR		69.7	1
* *	L.170.B.HEAR		117.3	1
* *	NPL.22B.HEAR		118.9	1
* *	MP28	A1	165.8	2
*P*. *crassamura*	PLRA-SFPUC.1	i	7.0	1
* *	TEVA-59B-ALRU.1	i	45.5	1
* *	SM-45B-FRCA.1	iib	89.2	1
* *	MA-85B-SOIL.1	iib	98.4	1
* *	PLRA-DRYSOIL1A.1	i	107.9	1
* *	SM-39B-FRCA.1	iib	124.6	1
* *	TEVA-326B-JUEF.1	i	164.0	2
* *	FOR-OUT-06B.1	iib	275.9	2
*P*. *lateralis*	PL-47		0.9	1
* *	PL-9		1.0	1
	Pl-28		2.1	1
	PL-34		2.4	1
	PL-54		57.3	1
	PL-31		102.3	1
	PL-25		273.8	2

The isolates of *P*. *cactorum*, *P*. *cinnamomi*, *P*. *cryptogea*, *P*. *megasperma*, *P*. *multivora*, *P*. *nemorosa* and *P*. *syringae* were all sensitive to phosphite as determined by the k-means clustering, and had EC50 values below 188 μg/mL (Table 4).

**Table 4:** The EC50 values of the *Pytophthora cactorum*, *P*. *cinnamomi*, *P*. *cryptogea*, *P*. *megasperma*, *P*. *multivora*, *P*. *nemorosa*, and *P*. *syringae* isolates which were all clustered in the more phosphite sensitive group (mean EC50 value of 33.8 μg/mL phosphite) in the K means analysis.

Species	Isolate	EC50
*P*. *cactorum*	7912.1	9.9
* *	7-HR.1	11.3
* *	PNPN-C-39FRCA.1	13.2
* *	10-SP.1	26.4
* *	MP19	26.4
* *	AKWA	27.6
* *	7-HP.1	30.8
* *	117R	113.6
*P*. *cinnamomi* A2	MC11	1.4
A2	P.6377 COFFEY	4.1
A2	P.3662 COFFEY	4.2
A1	P.6493 COFFEY	5.8
A1	P.2021 COFFEY	38.1
*P*. *cryptogea*	NPC-79B-MIAU.1	3.9
* *	ENPN122-DP-DIAU12.1	4.9
* *	ENPN80-B-DIAU10.2	5.4
* *	CCWN-295B-DIAU5.1	6.0
* *	MBP-B-DIAU1.1	7.8
* *	ENPN123-DP-DIAU13.2	7.9
* *	CCW-DP-DIAU-ROOTS.1	30.2
* *	MBP-B-DIAU4.1	50.8
*P*. *megasperma*	MBP-B-DIAU10.1	13.3
* *	MBP-DIAU5-DPSTEM.1	81.0
* *	MBP-DIAU4-DPSTEM.1	83.6
* *	MBP-DIAU10-DPSTEM.1	118.0
*P*. *multivora*	FOFU-C2-CETH.1	1.7
* *	NPC-47B-CETH.1	1.9
* *	MA-33B-FRCA.1	3.1
* *	MA-60B-FRCA.1	8.1
*P*. *nemorosa*	5104_Hansen22	0
* *	2052.2 _Hansen2	0.1
* *	2059.4 _Hansen6	0.4
* *	P.106	0.5
* *	P.114	0.6
* *	P.113	1.1
* *	1050_Hansen1	3.0
* *	P.115	27.6
*P*. *syringae*	SM15FEB_HOP	2.3
* *	SM15FEB_5CRP	6.9
* *	KDA_RT9	9.1
* *	MP-12	11.6
* *	SM15APR_B0V	25.8
* *	BSP2014_502	66.5
* *	MP-15	73.0

### Growth observations

Growth was ‘promoted’ on low concentrations of phosphite for five *P*. *crassamura*, one *P*. *cactorum*, one *P*. *cambivora*, two *P*. *lateralis*, two *P*. *syringae*, and seven *P*. *ramorum* isolates. This was generally observed as the mycelium growing less densely in response to the presence of phosphite and the culture having a larger diameter than that of the control.

## Discussion

Variation in phosphite tolerance of isolates within the same *Phytophthora* species is a concern for the future efficacy of phosphite treatments, as it shows adaptive evolution in isolates exposed to a novel selection pressure over a relatively short period of time. *Phytophthora* species have been shown to differ in their underlying tolerance to phosphite [20] and prolonged exposure to phosphite can lead to phosphite tolerant isolates of *Phytophthora* [4, 5, 21]. However, the identification of variability within genetically homogeneous and clonally reproducing taxa (such as lineages within species or entire homothallic species) is new and the result of fast adaptive evolutionary processes combined with differential history of exposure. In this case, we have identified several such cases (Figs 1 and 3 and Tables 2 and 3); for each one of them, tolerance can be used as a phenotypic marker of previous exposure to the chemical.

With a few exceptions (Iberia, New Zealand and Australia), phosphite is used only in plant production facilities, hence the presence of the trait in genotypes from the wild is the tell-tale sign that the genotype in question (or a close parental relative of the genotype) comes from a plant production facility where phosphite has been used. The use of this phenotypic marker has been recently adopted to reconstruct the likely (nursery) origin of *P*. *crassamura* isolates in the wild in California (Sims and Garbelotto, personal communication). However, there is a much more critical consequence of tolerance of phosphite in wild isolates that is exemplified for the first time by this study. The discovery that the EU1 lineage, found in Oregon forests, is phosphite tolerant raises concerns of the validity of employing phosphite as a primary disease control approach where EU1 may be present. The same appears to be true for the NA2 lineage if that were to be released in the wild. Further *in planta* testing will provide confidence in the efficacy of phosphite to control wild populations of EU1 lineage.

*Phytophthora ramorum* is a highly internationally regulated widespread pathogen present in ornamental nurseries worldwide and is also known to cause Sudden Oak Death in North America and Sudden Larch Death in Europe [7]. Isolates of the NA1 lineage of *P*. *ramorum* were variable in their tolerance to phosphite with two isolates being grouped in the more tolerant cluster in the K means analysis and the other four grouping in the more sensitive group (Fig 3 and Table 2). Therefore, multiple introductions of *P*. *ramorum* NA1 should be avoided because we may be introducing resistant individuals into a population that yet does not have it.

The NA2 *P*. *ramorum* isolates are from a nursery and it is likely they were previously exposed to phosphite. Because NA2 isolates are known only from nursery settings, it is not possible to determine how much of the apparent tolerance in the NA2 isolates may be due to a generalised underlying resistance at the lineage level, or if it may be due to adaptive evolution in response to phosphite. However, the fact that all NA2 isolates are associated with the ornamental plant industry [7, 22], combined with the finding that a random sample of NA2 isolates was entirely tolerant to phosphite, does suggest a “cause” (exposure to phosphite in nursery) and an “effect” (development of tolerance to phosphite).

All NA1 isolates were isolated from California bay laurel (*Umbellularia californica*) in the wild, but Croucher, Mascheretti (8) have shown that the entire NA1 population in California derives from a founder population in ornamental nurseries, hence they may have been exposed to the chemical at one point or another.

Intraspecific variation in phosphite sensitivity was also present among isolates of *P*. *cambivora*, *P*. *crassamura* and *P*. *lateralis* tested (Table 3). The hybrid nature of *P*. *cambivora* and associated ploidy variations may account for the variability in phosphite sensitivity observed (Table 3). There was no difference in the phosphite sensitivity of the two *P*. *crassamura* haplotypes i and iib (Table 3), but there was variation within each one of the two lineages tested. This finding suggests these species can also develop tolerance to phosphite and that a different history of exposure to phosphite may have occurred, e.g. some individuals may have been significantly exposed to the chemical, while others may have not. This is not surprising, because all three species are known to be present in commercial nurseries [23]. Like *P*. *ramorum*, *P*. *crassamura* and *P*. *lateralis* are not known to be sexually outcrossing, thus we conclude that *P*. *ramorum*, *P*. *lateralis* (a close relative of *P*. *ramorum*), and *P*. *crassamura* may develop tolerance to phosphite even in the absence of sexually induced recombination. Changes in ploidy and in chromosome copy numbers have been associated with the insurgence of tolerance to chemicals [12] and have reported to occur in *P*. *ramorum* [9]. We suggest this genomic mechanism may be at least in part responsible for this important phenotypic trait.

At low phosphite concentrations there may be an enhancement in the linear growth of *Phytophthora* species on agar and this is expected as it may be a survival strategy (Fig 1).

In this situation the use of dry weight or optical density measurements would have more accurately captured the true inhibition because density could be accounted for. It is also relevant that the growth was promoted on 10% V8 media because it is probably easier for the *Phytophthora* to alter growth when in nutrient and phosphate rich media as it is not phosphate starved.

Seven of the species tested appear to be sensitive to phosphite as all isolates were clustered in the sensitive group of the K means analysis (Table 4); of course results could be different if the number of isolates tested for each species were to increase. As a species, *P*. *cinnamomi* has previously been reported as sensitive to phosphite [20], however after prolonged exposure isolates can develop tolerance [4, 5]. No significant difference in the phosphite tolerance was observed between the A1 and A2 mating types in the current study or previous studies [20, 24], and no differences were observed in this study given the sample of *P*. *cinnamomi* that was tested. It has been shown previously that *P*. *ramorum* was generally less sensitive to phosphite [25] compared to *P*. *cinnamomi* [24], and the results from the current study support this previous finding (Figs 1 and 2). Phosphite is used abundantly in avocado orchards to control phytophthora root rot caused by *P*. *cinnamomi* and recovery of trees infected with *P*. *cinnamomi* upon treatment with phosphite has been reported [26]. In comparison the direct fungistatic effects of phosphite on *P*. *ramorum* may be modest [25], and because of this, phosphite is always recommended as preventive and not therapeutic treatments against *P*. *ramorum* [27]. Higher average tolerance values can be associated either with species that appear to have a generalized tolerance across all individuals or with species that include some individuals characterized by higher tolerance.

## Conclusions

Seven of the 11 species tested for phosphite tolerance were found to be sensitive and without intraspecific variation based on the sample size tested for each species, however intraspecific variability in tolerance was observed in the remaining four species. Variability found within two individual clonally reproducing lineages of *P*. *ramorum* and within two lineages of *P*. *crassamura* suggest the selection pressure of phosphite has allowed individual isolates to adapt and evolve to gain an increased tolerance to phosphite at least twice. The interesting finding that all NA2 isolates from ornamental plant nurseries were relatively tolerant to phosphite, suggests that chemical treatments in these settings may be responsible for the development of this adaptive trait.

## Supporting information

S1 Table Linear growth measurements of the *Phytophthora* cultures 5 days after inoculation. Treatment is phosphite concentration (μg/mL). Blank square is no measurement. *Phytophthora megasperma* isolate MBP-B-DIAU10.1 outgrew the control plates on day 5 so
(PDF)

S2 Table Linar growth measurements of the *Phytophthora lateralis* cultures 7 days after inoculation. Treatment is phosphite concentration (μg/mL). Blank square is no measurement.
(PDF)
