# Supplementary material for: Evidence for rapid adaptive evolution of tolerance to chemical treatments in Phytophthora species and its practical implications
Source: PLoS One. 2018 Dec 10;13(12):e0208961. doi: 10.1371/journal.pone.0208961 (PMC6287812; doi:10.1371/journal.pone.0208961)
Supplement: S1 Table — Treatment is phosphite concentration (μg/mL). Blank square is no measurement. Phytophthora megasperma isolate MBP-B-DIAU10.1 outgrew the control plates on day 5 so (PDF) [file pone.0208961.s001.pdf]

				Diameter (mm) including 5mm inoculum plug					
Species	Isolate name	Isolate number	Treatment (µg/mL phosphite)	rep 1		rep 2		rep 3	
P. cryptogea	MBP-B-DIAU1.1	1	0	56	56	52	54	54	55
P. cryptogea	MBP-B-DIAU1.1	1	15	28	28	29	29	28	28
P. cryptogea	MBP-B-DIAU1.1	1	40	23	24	22	22	22	22
P. cryptogea	MBP-B-DIAU1.1	1	80	23.5	24	23	23	23	23
P. cryptogea	MBP-B-DIAU1.1	1	200	20	21	20	19	20	20
P. cryptogea	MBP-B-DIAU1.1	1	500	17	16	14	17	17	14
P. cryptogea	CCWN-295B-DIAU5.1	2	0	59	60	57	58	63	63
P. cryptogea	CCWN-295B-DIAU5.1	2	15	28	30	29	27	30	31
P. cryptogea	CCWN-295B-DIAU5.1	2	40	26	26	25	25	24	24
P. cryptogea	CCWN-295B-DIAU5.1	2	80	25	25	22	23	22	23
P. cryptogea	CCWN-295B-DIAU5.1	2	200	22	21	22	21	20	20
P. cryptogea	CCWN-295B-DIAU5.1	2	500	18	16	16	14	15	15
P. cryptogea	MBP-B-DIAU4.1	3	0	54	50	50	50	51	51
P. cryptogea	MBP-B-DIAU4.1	3	15	37	35	35	35	34	36
P. cryptogea	MBP-B-DIAU4.1	3	40	31	30	31	31	30	31
P. cryptogea	MBP-B-DIAU4.1	3	80	24	23	23	23	22	22
P. cryptogea	MBP-B-DIAU4.1	3	200	20	21	21	21	21	20
P. cryptogea	MBP-B-DIAU4.1	3	500	15	17	16	17	10	15
P. cryptogea	CCW-DP-DIAU-ROOTS.1	4	0	53	52	54	53	54	55
P. cryptogea	CCW-DP-DIAU-ROOTS.1	4	15	33	35	33	33	32	32
P. cryptogea	CCW-DP-DIAU-ROOTS.1	4	40	28	28	27	27	27	27
P. cryptogea	CCW-DP-DIAU-ROOTS.1	4	80	24	25	23	24	26	26
P. cryptogea	CCW-DP-DIAU-ROOTS.1	4	200	21	20	21	21	21	21
P. cryptogea	CCW-DP-DIAU-ROOTS.1	4	500	18	18	17	17	17	18
P. cryptogea	NPC-79B-MIAU.1	5	0	63	67	64	64	62	63
P. cryptogea	NPC-79B-MIAU.1	5	15	27	28	26	26	27	27
P. cryptogea	NPC-79B-MIAU.1	5	40	22	22.5	20	21	21	20
P. cryptogea	NPC-79B-MIAU.1	5	80	13	12	12	12	13	13
P. cryptogea	NPC-79B-MIAU.1	5	200	12	11	11	12	11	11
P. cryptogea	NPC-79B-MIAU.1	5	500	5	5.5	5	5	5	5
P. cryptogea	ENPN122-DP-DIAU12.1	6	0	56	54	55	55	58	57
P. cryptogea	ENPN122-DP-DIAU12.1	6	15	27	27	25	26	26	25
P. cryptogea	ENPN122-DP-DIAU12.1	6	40	19.5	19.5	17	18	18	18
P. cryptogea	ENPN122-DP-DIAU12.1	6	80	12	12	9	8	12	11.5
P. cryptogea	ENPN122-DP-DIAU12.1	6	200	11.5	11.5	11	11	12	12
P. cryptogea	ENPN122-DP-DIAU12.1	6	500	5.5	5.5	5	5.5	5	5
P. cryptogea	ENPN123-DP-DIAU13.2	7	0	55	56	47	50	52	53
P. cryptogea	ENPN123-DP-DIAU13.2	7	15	27	28	25	26	25	26
P. cryptogea	ENPN123-DP-DIAU13.2	7	40	22	21	19	20	18	18
P. cryptogea	ENPN123-DP-DIAU13.2	7	80	12	12	11	11	11	11.5
P. cryptogea	ENPN123-DP-DIAU13.2	7	200	12	12	11	10.5	10.5	10
P. cryptogea	ENPN123-DP-DIAU13.2	7	500	5.5	5	5.5	5	5.5	5.5
P. cryptogea	ENPN80-B-DIAU10.2	8	0	54	55	52	53	56	57
P. cryptogea	ENPN80-B-DIAU10.2	8	15	26	26	25	25	25	24
P. cryptogea	ENPN80-B-DIAU10.2	8	40	20	20	18	18	19	18
P. cryptogea	ENPN80-B-DIAU10.2	8	80	11	11	11	10	11	10

				Diameter (mm) including 5mm inoculum plug					
**Species**	**Isolate name**	**Isolate number**	**Treatment (µg/mL phosphite)**	**rep 1**		**rep 2**		**rep 3**	
P. cryptogea	ENPN80-B-DIAU10.2	8	200	9	9	8	8	9	9
P. cryptogea	ENPN80-B-DIAU10.2	8	500	5	5	5	5	5	5
P. multivora	FOFU-C2-CETH.1	9	0	50	48	46	48	47	45
P. multivora	FOFU-C2-CETH.1	9	15	18	19	17	18	18	18
P. multivora	FOFU-C2-CETH.1	9	40	15	15	13	14	15	17
P. multivora	FOFU-C2-CETH.1	9	80	9	7	9	9	8	8
P. multivora	FOFU-C2-CETH.1	9	200	7	5.5	6	6	6	6
P. multivora	FOFU-C2-CETH.1	9	500	5	5	5	5	5	5
P. multivora	NPC-47B-CETH.1	10	0	51	53	47	47	46	47
P. multivora	NPC-47B-CETH.1	10	15	20	20	18	18	22	23
P. multivora	NPC-47B-CETH.1	10	40	13	11	12	12	14	14
P. multivora	NPC-47B-CETH.1	10	80	7	7	7	7	7	8
P. multivora	NPC-47B-CETH.1	10	200	5.5	5	5	5.5	6	5
P. multivora	NPC-47B-CETH.1	10	500	5	5	5	5	5	5
P. multivora	MA-33B-FRCA.1	11	0	55	54	51	52	52	51
P. multivora	MA-33B-FRCA.1	11	15	22	21	22	22	23	23
P. multivora	MA-33B-FRCA.1	11	40	19	18	18	19	18	18
P. multivora	MA-33B-FRCA.1	11	80	13	13	14	14	14	13
P. multivora	MA-33B-FRCA.1	11	200	10	10.5	10	11	11	10
P. multivora	MA-33B-FRCA.1	11	500	9	7	8	6.5	7	7
P. multivora	MA-60B-FRCA.1	12	0	58	58	56	54	55	55
P. multivora	MA-60B-FRCA.1	12	15	27	30	33	35	26	32
P. multivora	MA-60B-FRCA.1	12	40	16	16	15	17	15	16
P. multivora	MA-60B-FRCA.1	12	80	13	14	13	17	14	13
P. multivora	MA-60B-FRCA.1	12	200	10	12	10	11	11	11
P. multivora	MA-60B-FRCA.1	12	500	8	5	7	5	5	5
P. crassamura	PLRA-SFPUC.1	13	0	48	47	47	46	47	47
P. crassamura	PLRA-SFPUC.1	13	15	40	39	39	38	38	38
P. crassamura	PLRA-SFPUC.1	13	40	28	30	28	27	29	30
P. crassamura	PLRA-SFPUC.1	13	80	24	24	27	27	23	24
P. crassamura	PLRA-SFPUC.1	13	200	23	23	21	21	19	20
P. crassamura	PLRA-SFPUC.1	13	500	15	16	14	13	12	13
P. crassamura	PLRA-DRYSOIL1A.1	14	0	39	41	42	41	39	39
P. crassamura	PLRA-DRYSOIL1A.1	14	15	41	44	39	40	40	40
P. crassamura	PLRA-DRYSOIL1A.1	14	40	31	33	30	29	31	32
P. crassamura	PLRA-DRYSOIL1A.1	14	80	24.5	24	15	16	19	20
P. crassamura	PLRA-DRYSOIL1A.1	14	200	18	18.5	19	19	19	19
P. crassamura	PLRA-DRYSOIL1A.1	14	500	12	12	13	13	10	10
P. crassamura	TEVA-59B-ALRU.1	15	0	48	48	47	48	48	48
P. crassamura	TEVA-59B-ALRU.1	15	15	37	37	37	38	37	38
P. crassamura	TEVA-59B-ALRU.1	15	40	33	32	29	31	25	26
P. crassamura	TEVA-59B-ALRU.1	15	80	15	15	15	15	15	15
P. crassamura	TEVA-59B-ALRU.1	15	200	15.5	15	15	15	15	14
P. crassamura	TEVA-59B-ALRU.1	15	500	11.5	13	10	9	11	9
P. crassamura	TEVA-326B-JUEF.1	16	0	34	34	33	33	35	37
P. crassamura	TEVA-326B-JUEF.1	16	15	39	40	39	40	41	40

				Diameter (mm) including 5mm inoculum plug					
**Species**	**Isolate name**	**Isolate number**	**Treatment (µg/mL phosphite)**	**rep 1**		**rep 2**		**rep 3**	
P. crassamura	TEVA-326B-JUEF.1	16	40	33	32	32	33	32	31
P. crassamura	TEVA-326B-JUEF.1	16	80	20	18	18	17	18	18
P. crassamura	TEVA-326B-JUEF.1	16	200	20	20	19	20	20	20
P. crassamura	TEVA-326B-JUEF.1	16	500	13	14	13	13	11	12
P. crassamura	SM-45B-FRCA.1	17	0	35	35	34	35	34	34
P. crassamura	SM-45B-FRCA.1	17	15	36	35	37	34	38	35
P. crassamura	SM-45B-FRCA.1	17	40	29	28	26	27	26	27
P. crassamura	SM-45B-FRCA.1	17	80	17	17	12	12	17	15
P. crassamura	SM-45B-FRCA.1	17	200	13	13	12	13	12	12
P. crassamura	SM-45B-FRCA.1	17	500	10	9	10	10	9	9
P. crassamura	SM-39B-FRCA.1	18	0	28	30	28	29	31	31
P. crassamura	SM-39B-FRCA.1	18	15	38	38	35	38	35	36
P. crassamura	SM-39B-FRCA.1	18	40	27	26	28	28	25	26
P. crassamura	SM-39B-FRCA.1	18	80	17	16	16	15	16	18
P. crassamura	SM-39B-FRCA.1	18	200	11	12	13.5	13	12	13
P. crassamura	SM-39B-FRCA.1	18	500	10	9	7	8	10	9
P. crassamura	FOR-OUT-06B.1	19	0	24	23	24	24	24	24
P. crassamura	FOR-OUT-06B.1	19	15	39	40	41	41	40	40
P. crassamura	FOR-OUT-06B.1	19	40	33	33	30	30	30	30
P. crassamura	FOR-OUT-06B.1	19	80	21	22	21	21	22	22.5
P. crassamura	FOR-OUT-06B.1	19	200	17	18	18	18	18	18
P. crassamura	FOR-OUT-06B.1	19	500	11	11	10	10	10	10
P. crassamura	MA-85B-SOIL.1	20	0	33	33	34	37	36	36
P. crassamura	MA-85B-SOIL.1	20	15	32	32	35	35	32	31
P. crassamura	MA-85B-SOIL.1	20	40	25	26	25	27	27	26
P. crassamura	MA-85B-SOIL.1	20	80	18	18	17	17	16	17
P. crassamura	MA-85B-SOIL.1	20	200	16	14	14	15	15	16
P. crassamura	MA-85B-SOIL.1	20	500	12	13	12	13	12	12
P. megasperma	MBP-DIAU10-DPSTEM.1	21	0	48	45	50	48	48	48
P. megasperma	MBP-DIAU10-DPSTEM.1	21	15	44	45	43	43	44	43
P. megasperma	MBP-DIAU10-DPSTEM.1	21	40	35	36	35	35	36	37
P. megasperma	MBP-DIAU10-DPSTEM.1	21	80	28	28	22	21	25	27
P. megasperma	MBP-DIAU10-DPSTEM.1	21	200	29	27	24	25	25	23
P. megasperma	MBP-DIAU10-DPSTEM.1	21	500	12	14	16	15	14	15
P. megasperma	MBP-DIAU5-DPSTEM.1	22	0	50	49	50	52	49	50
P. megasperma	MBP-DIAU5-DPSTEM.1	22	15	43	43	34	34	42	43
P. megasperma	MBP-DIAU5-DPSTEM.1	22	40	36	35	25	24	36	33
P. megasperma	MBP-DIAU5-DPSTEM.1	22	80	25	25	26	25	25	25
P. megasperma	MBP-DIAU5-DPSTEM.1	22	200	26	26	24	23	25	24
P. megasperma	MBP-DIAU5-DPSTEM.1	22	500	15	15	15	15	12	12
P. megasperma	MBP-DIAU4-DPSTEM.1	23	0	49	49	51	48	48	52
P. megasperma	MBP-DIAU4-DPSTEM.1	23	15	41	44	42	43	42	43
P. megasperma	MBP-DIAU4-DPSTEM.1	23	40	36	36	33	35	35	35
P. megasperma	MBP-DIAU4-DPSTEM.1	23	80	24	26	24	24	24	23
P. megasperma	MBP-DIAU4-DPSTEM.1	23	200	22	24	19	18	19	20
P. megasperma	MBP-DIAU4-DPSTEM.1	23	500	11	12	13	14	14	13

				Diameter (mm) including 5mm inoculum plug					
**Species**	**Isolate name**	**Isolate number**	**Treatment (µg/mL phosphite)**	**rep 1**		**rep 2**		**rep 3**	
P. megasperma	MBP-B-DIAU10.1	24	0	90	90	90	90	90	90
P. megasperma	MBP-B-DIAU10.1	24	15	82	82	76	76	80	80
P. megasperma	MBP-B-DIAU10.1	24	40	57	45	50	51	46	46
P. megasperma	MBP-B-DIAU10.1	24	80	36	37	38	40	36	36
P. megasperma	MBP-B-DIAU10.1	24	200	16	15	15	15	15	16
P. megasperma	MBP-B-DIAU10.1	24	500	10	9	9	9	10	10
P. cactorum	7-HR.1	25	0	43	43	40	41	45	45
P. cactorum	7-HR.1	25	15	24	24	23	22	24	26
P. cactorum	7-HR.1	25	40	19	19	19	20	19	18
P. cactorum	7-HR.1	25	80	18	19	18	19	18	18
P. cactorum	7-HR.1	25	200	17	17	18	18	17	18
P. cactorum	7-HR.1	25	500	12	13	13	13	13	13
P. cactorum	7-HP.1	26	0	48	50	47	48	52	52
P. cactorum	7-HP.1	26	15	34	35	35	34	33	33
P. cactorum	7-HP.1	26	40	28	29	28	28	28	28
P. cactorum	7-HP.1	26	80	15	14	13	11	11	12
P. cactorum	7-HP.1	26	200	16	16	16	16	16	17
P. cactorum	7-HP.1	26	500	12	12	12	11	9	11
P. cactorum	PNPN-C-39FRCA.1	27	0	12	13	44	44	43	42
P. cactorum	PNPN-C-39FRCA.1	27	15	26	27	26	26	22	23
P. cactorum	PNPN-C-39FRCA.1	27	40	22	21	21	22	21	22
P. cactorum	PNPN-C-39FRCA.1	27	80	9	8	8	8	17	18
P. cactorum	PNPN-C-39FRCA.1	27	200	16	17	16	17	16	16
P. cactorum	PNPN-C-39FRCA.1	27	500	10	11	11	10.5	10	10
P. cactorum	10-SP.1	28	0	44	43	45	46	44	44
P. cactorum	10-SP.1	28	15	32	30	31	32	29	31
P. cactorum	10-SP.1	28	40	22	24	23	23	24	25
P. cactorum	10-SP.1	28	80	7	7	9	8	14	13
P. cactorum	10-SP.1	28	200	13	11	12	12	11	12
P. cactorum	10-SP.1	28	500	6	6	6	7	6	6.5
P. cactorum	AKWA	29	0	30	31	32	34	36	36
P. cactorum	AKWA	29	15	28	28	25	26	19	19
P. cactorum	AKWA	29	40	16	17	20	20	20	19
P. cactorum	AKWA	29	80	7	7	7	7	5	5
P. cactorum	AKWA	29	200	9	9	9	9	10	8
P. cactorum	AKWA	29	500	5	5	5	5	5	5
P. cactorum	7912.1	30	0	43	42	44	43	44	43
P. cactorum	7912.1	30	15	23	24	23	23	23	23
P. cactorum	7912.1	30	40	21	21	20	20	20	21
P. cactorum	7912.1	30	80	8	8.5	8	8	8	8
P. cactorum	7912.1	30	200	9	9	13	14	12.5	13
P. cactorum	7912.1	30	500	8	8	7	7	7	8
P. cactorum	117R	31	0	25	27	22	24	25	28
P. cactorum	117R	31	15	28	29	28	29	30	29
P. cactorum	117R	31	40	27	25	24	25	25	25
P. cactorum	117R	31	80	10	10	9	10	11	12

				Diameter (mm) including 5mm inoculum plug					
Species	Isolate name	Isolate number	Treatment (µg/mL phosphite)	rep 1		rep 2		rep 3	
P. cactorum	117R	31	200	11	13	13	12	12	12
P. cactorum	117R	31	500	8	9	10	7	5	5
P. cactorum	MP19	32	0	28	27	28	28	30	30
P. cactorum	MP19	32	15	21	22	19	19	20	19
P. cactorum	MP19	32	40	17	16	16	16	16	16
P. cactorum	MP19	32	80	8	7	8	9	9	9
P. cactorum	MP19	32	200	8	8.5	8	9	8	9
P. cactorum	MP19	32	500	5	5	5	5	5	5
P. cambivora	MP21	33	0	28	29	28	28	28	28
P. cambivora	MP21	33	15	16	16	14	14	17	17
P. cambivora	MP21	33	40	14	14	13	13	14	15
P. cambivora	MP21	33	80	10	9	10	11	7	8
P. cambivora	MP21	33	200	18	20	19	18	14	15
P. cambivora	MP21	33	500	14	14	13	13	15	14
P. cambivora	MP28	34	0	27	28	26	27	25	24
P. cambivora	MP28	34	15	21	20	14	15	15	17
P. cambivora	MP28	34	40	18	18	17	17	17	17
P. cambivora	MP28	34	80	18	18	12	11	13	11
P. cambivora	MP28	34	200	7	10	9	5	10	10
P. cambivora	MP28	34	500	5	5	5	5	5	5
P. cambivora	MP28	35	0	15	15	15	12	14	14
P. cambivora	MP28	35	15	20	19	17	17	17	18
P. cambivora	MP28	35	40	15	14	15	14	16	17
P. cambivora	MP28	35	80	8	8	8	8	7	7
P. cambivora	MP28	35	200	8	7	11	9	12	11
P. cambivora	MP28	35	500	9	9	5	7	6	8
P. cambivora	L.170.B.HEAR	36	0	42	43	44	44	42	45
P. cambivora	L.170.B.HEAR	36	15	41	41	40	40	40	40
P. cambivora	L.170.B.HEAR	36	40	38	34	32	32	32	32
P. cambivora	L.170.B.HEAR	36	80	21	22	21	20	18	18
P. cambivora	L.170.B.HEAR	36	200	21	22	22	22	22	21
P. cambivora	L.170.B.HEAR	36	500	17	17	15	15	17	15
P. cambivora	FOR.61B.HEAR	37	0	41	43	40	40	42	44
P. cambivora	FOR.61B.HEAR	37	15	40	42	39	3	38	39
P. cambivora	FOR.61B.HEAR	37	40	27	27	27	28	29	30
P. cambivora	FOR.61B.HEAR	37	80	17	17	18	18	15	16
P. cambivora	FOR.61B.HEAR	37	200	21	16	16	17	14	15
P. cambivora	FOR.61B.HEAR	37	500	16	18	14	16	13	15
P. cambivora	NPL.22B.HEAR	38	0	23	25	26	26	27	28
P. cambivora	NPL.22B.HEAR	38	15	25	24	25	25	25	26
P. cambivora	NPL.22B.HEAR	38	40	19	19	18	18	13	13
P. cambivora	NPL.22B.HEAR	38	80	12	13	13	14	15	14
P. cambivora	NPL.22B.HEAR	38	200	17	18	17	18	15	14
P. cambivora	NPL.22B.HEAR	38	500	10	9	13	10	11	10
P. cinnamomi	MC11	39	0	39	42	40	41	42	42
P. cinnamomi	MC11	39	15	20	19	18	18	18	19

				Diameter (mm) including 5mm inoculum plug					
**Species**	**Isolate name**	**Isolate number**	**Treatment (µg/mL phosphite)**	**rep 1**		**rep 2**		**rep 3**	
P. cinnamomi	MC11	39	40			10	10	9	10
P. cinnamomi	MC11	39	80	5	5	5	5	5	5
P. cinnamomi	MC11	39	200	5	5	5	5	5	5
P. cinnamomi	MC11	39	500	5	5	5	5	5	5
P. cinnamomi	P.2021 COFFEY	40	0	34	32	29	30	33	38
P. cinnamomi	P.2021 COFFEY	40	15	34	33	27	26	23	24
P. cinnamomi	P.2021 COFFEY	40	40	20	18	17	17	17	18
P. cinnamomi	P.2021 COFFEY	40	80	9	9	10	10	9	9
P. cinnamomi	P.2021 COFFEY	40	200	7	7	6	7	7	7
P. cinnamomi	P.2021 COFFEY	40	500	5	5	5	5	5	5
P. cinnamomi	P.3662 COFFEY	41	0	50	48	45	44	44	43
P. cinnamomi	P.3662 COFFEY	41	15	20	20	21	21	20	20
P. cinnamomi	P.3662 COFFEY	41	40	18	18	13	14	15	15
P. cinnamomi	P.3662 COFFEY	41	80	9	9.5	8	8.5	9.5	9.5
P. cinnamomi	P.3662 COFFEY	41	200	5	5	5	5	5	5
P. cinnamomi	P.3662 COFFEY	41	500	5	5	5	5	5	5
P. cinnamomi	P.6377 COFFEY	42	0	38	8	34	34	38	40
P. cinnamomi	P.6377 COFFEY	42	15	15	14	14	14	14	14
P. cinnamomi	P.6377 COFFEY	42	40	12	12	11	11	11	10
P. cinnamomi	P.6377 COFFEY	42	80	10	9	8	8	9	9
P. cinnamomi	P.6377 COFFEY	42	200	5.5	5.5	5.5	5.5	5.5	5.5
P. cinnamomi	P.6377 COFFEY	42	500	5	5	5	5	5	5
P. cinnamomi	P.6493 COFFEY	43	0	55	57	55	53	57	57
P. cinnamomi	P.6493 COFFEY	43	15	27	27	26	26	26	25
P. cinnamomi	P.6493 COFFEY	43	40	17	17	18	17	19	18
P. cinnamomi	P.6493 COFFEY	43	80	18	18	10	10	10	10
P. cinnamomi	P.6493 COFFEY	43	200	5	5	6.5	7	6.5	6.5
P. cinnamomi	P.6493 COFFEY	43	500	5	5	5	5	5	5
P. nemorosa	P.106	44	0	53	53	53	53	52	51
P. nemorosa	P.106	44	15	19	18	18	18	17	18
P. nemorosa	P.106	44	40	12	13	12	12	11	10
P. nemorosa	P.106	44	80	9	8	8	7	8	8
P. nemorosa	P.106	44	200	5.5	5	5	5.5	5	5
P. nemorosa	P.106	44	500	5	5	5	5	5	5
P. nemorosa	P.113	45	0	51	52	50	50	53.5	53
P. nemorosa	P.113	45	15	20	19	17	18	17	17
P. nemorosa	P.113	45	40	14	15	19	18	13	13
P. nemorosa	P.113	45	80	11	10	10	9	10	10
P. nemorosa	P.113	45	200	6	6.5	5.5	6	7	6
P. nemorosa	P.113	45	500	6	5	5	5	5	5
P. nemorosa	P.114	46	0	54	53	48	48	52	53
P. nemorosa	P.114	46	15	17	17	20	16	18	17
P. nemorosa	P.114	46	40	13	13	14	13	12	13
P. nemorosa	P.114	46	80	7	7	7	7	7	7
P. nemorosa	P.114	46	200	5	5	5	5	5	5
P. nemorosa	P.114	46	500	5	5	5	5	5	5

				Diameter (mm) including 5mm inoculum plug					
**Species**	**Isolate name**	**Isolate number**	**Treatment (µg/mL phosphite)**	**rep 1**		**rep 2**		**rep 3**	
P. nemorosa	P.115	47	0	14	14	8	10	17	17
P. nemorosa	P.115	47	15	13	13	11	11	13	13
P. nemorosa	P.115	47	40	8	7.5	7	9	8	9
P. nemorosa	P.115	47	80	7	7	7	7	7	7
P. nemorosa	P.115	47	200	5	5	5	5	5	5
P. nemorosa	P.115	47	500	5	5	5	5	5	5
P. nemorosa	1050 Hansen1	48	0	44	44	44	43	45	46
P. nemorosa	1050 Hansen1	48	15	21	21	19	19	20	20
P. nemorosa	1050 Hansen1	48	40	13	13	11.5	12.5	12	13
P. nemorosa	1050 Hansen1	48	80	9	10	9	10	9	9
P. nemorosa	1050 Hansen1	48	200	7	6.5	7	6	6	6
P. nemorosa	1050 Hansen1	48	500	5	5	5	5	5	5
P. nemorosa	2052.2 Hansen2	49	0	57	57	57	58	55	54
P. nemorosa	2052.2 Hansen2	49	15	25	23	15	14	13	13
P. nemorosa	2052.2 Hansen2	49	40	12	11	10	11	10	11
P. nemorosa	2052.2 Hansen2	49	80	6	6	5.5	5	5.5	5.5
P. nemorosa	2052.2 Hansen2	49	200	5	5	5	5	5	5
P. nemorosa	2052.2 Hansen2	49	500	5	5	5	5	5	5
P. nemorosa	2059.4 Hansen6	50	0	54	56	55	56	54	54
P. nemorosa	2059.4 Hansen6	50	15	19	18	17	18	18	18
P. nemorosa	2059.4 Hansen6	50	40	12	12	10	11	12	12
P. nemorosa	2059.4 Hansen6	50	80	9	9	9	9	8	8
P. nemorosa	2059.4 Hansen6	50	200	5	5	5	5	5	5
P. nemorosa	2059.4 Hansen6	50	500	5	5	5	5	5	5
P. nemorosa	5104 Hansen22	51	0	39	39	10	10	40	39
P. nemorosa	5104 Hansen22	51	15	12	12	12	12	11	12
P. nemorosa	5104 Hansen22	51	40	7	7	7	7	7	7
P. nemorosa	5104 Hansen22	51	80	7	7	6	6	6	6
P. nemorosa	5104 Hansen22	51	200	5	5	5	5	5	5
P. nemorosa	5104 Hansen22	51	500	5	5	5	5	5	5
P. lateralis	PL-9	52	0	18	18	16	16	16	17
P. lateralis	PL-9	52	15	9	9	9.5	10	10	10
P. lateralis	PL-9	52	40	6	6	6	6	6	6
P. lateralis	PL-9	52	80	6	7	7	6.5	6	7
P. lateralis	PL-9	52	200	6	6	7	7	7	7
P. lateralis	PL-9	52	500	5.5	5.5	5	5.5	5	5
P. lateralis	PL-25	53	0	6	6	6	6	7	7
P. lateralis	PL-25	53	15	7.5	8	8	7.5	8	7
P. lateralis	PL-25	53	40	7	8	7	7.5	7	8
P. lateralis	PL-25	53	80	6	7	7	6	7	7
P. lateralis	PL-25	53	200	7	7	7	7	6.5	7
P. lateralis	PL-25	53	500	5	5.5	5	5	5	6
P. lateralis	Pl-28	54	0	16	16	15	16	16	17
P. lateralis	Pl-28	54	15	9	9	9	9.5	9.5	9
P. lateralis	Pl-28	54	40	5	5.5	5	5.5	5.5	6
P. lateralis	Pl-28	54	80	6	5.5	5.5	5	6	6

				Diameter (mm) including 5mm inoculum plug					
Species	Isolate name	Isolate number	Treatment (µg/mL phosphite)	rep 1		rep 2		rep 3	
P. lateralis	Pl-28	54	200	5.5	5	5	5	5	5
P. lateralis	Pl-28	54	500	5	5	5	5	5	5
P. lateralis	PL-31	55	0	9	8	7	8	7	7
P. lateralis	PL-31	55	15	8	8	8	7.5	8	8
P. lateralis	PL-31	55	40	8	8	8	8	7.5	7.5
P. lateralis	PL-31	55	80	7	7.5	7	7	6	6
P. lateralis	PL-31	55	200	7	7	6.5	7	7	8
P. lateralis	PL-31	55	500	5.5	5.5	5.5	5	5	5
P. lateralis	PL-34	56	0	15	15	14	14	14	14
P. lateralis	PL-34	56	15	9	9	9	8	9	9
P. lateralis	PL-34	56	40	6	6	6	6	6	6
P. lateralis	PL-34	56	80	6	6.5	6	6	6.5	6
P. lateralis	PL-34	56	200	6	6	6	6	6	6
P. lateralis	PL-34	56	500	5	5	5	5.5	5	5.5
P. lateralis	PL-47	57	0	11.5	11	9	10		
P. lateralis	PL-47	57	15	8	8	7	7		
P. lateralis	PL-47	57	40	7	5	6	7		
P. lateralis	PL-47	57	80	6	7.5	7	7		
P. lateralis	PL-47	57	200	7	6	7	7		
P. lateralis	PL-47	57	500	5.5	5.5	7	6		
P. lateralis	PL-54	58	0	6	6	6	6	6	5
P. lateralis	PL-54	58	15	6	6	6	6	6.5	6.5
P. lateralis	PL-54	58	40	6.5	6	6	6	6	6
P. lateralis	PL-54	58	80	5	5	5	5	5	5
P. lateralis	PL-54	58	200	5	5	5	5	5	5
P. lateralis	PL-54	58	500	5	5	5	5	5	5
P. syringae	MP-12	59	0	34	33	33	33	34	33
P. syringae	MP-12	59	15	21	21	21	20	18	18
P. syringae	MP-12	59	40	18	16	15	17	14	16
P. syringae	MP-12	59	80	16	16	16	15	15	15
P. syringae	MP-12	59	200	15	13	19	18	15	13
P. syringae	MP-12	59	500	14	14	13	13	13	14
P. syringae	MP-15	60	0	26	26	24	25	25	26
P. syringae	MP-15	60	15	20	18	19	19	20	19
P. syringae	MP-15	60	40	13	15	13	14	16	14
P. syringae	MP-15	60	80	17	18	19	17	17	18
P. syringae	MP-15	60	200	13	14	13	14	16	15
P. syringae	MP-15	60	500	9	10	8	8	9	7
P. ramorum	SI-556	61	0	17	17	15	16	18	18
P. ramorum	SI-556	61	15	24	24	22	22	22	22
P. ramorum	SI-556	61	40	20	20	21	21	17	18
P. ramorum	SI-556	61	80	10	10	10	9.5	10	10.5
P. ramorum	SI-556	61	200	12	11	10	10	11	11
P. ramorum	SI-556	61	500	8	9	8	9	8	8
P. ramorum	SI-592	62	0	25	25	9	8	11	12
P. ramorum	SI-592	62	15	25	24	22	24	23	23

				Diameter (mm) including 5mm inoculum plug					
Species	Isolate name	Isolate number	Treatment (µg/mL phosphite)	rep 1		rep 2		rep 3	
P. ramorum	SI-592	62	40	18	20	20	19	19	19
P. ramorum	SI-592	62	80	10	11	11	11	10	11
P. ramorum	SI-592	62	200	8	9	10	9.5	9	10
P. ramorum	SI-592	62	500	9	9	8	8.5	9	9
P. ramorum	SI-595	63	0	19	17	20	20	5	5
P. ramorum	SI-595	63	15	20	22	20	22	21	22
P. ramorum	SI-595	63	40	20	18	19	18	17	20
P. ramorum	SI-595	63	80	8	8	7	7.5	7	7
P. ramorum	SI-595	63	200	8	8	8	8	9	10
P. ramorum	SI-595	63	500	7	7	6.5	6	7	7
P. ramorum	MR-59	64	0	20	19	20	19	22	22
P. ramorum	MR-59	64	15	24	24	24	24	25	26
P. ramorum	MR-59	64	40	20	18	14	15	17	16
P. ramorum	MR-59	64	80	12	13	14	14	13	14
P. ramorum	MR-59	64	200	13	14	14	14	13	15
P. ramorum	MR-59	64	500	10	10	10	10	9	9
P. ramorum	MR-64	65	0	12	10	10	9	10	11
P. ramorum	MR-64	65	15	17	17.5	17	19	17	18
P. ramorum	MR-64	65	40	26	23	22	23	23	23
P. ramorum	MR-64	65	80	6.5	8			8	9
P. ramorum	MR-64	65	200	11	11	11	12	11	15
P. ramorum	MR-64	65	500	9	9	9	7	9	9
P. ramorum	MR-69	66	0	19	18	19	19	19	19
P. ramorum	MR-69	66	15	13	13	16	16	16	17
P. ramorum	MR-69	66	40	19	19	20	20	19	20
P. ramorum	MR-69	66	80	15	15	13	14	13	12
P. ramorum	MR-69	66	200	16	14	15	15	14	15
P. ramorum	MR-69	66	500	10	8	9	10	10	9
P. ramorum	MR-88	67	0	26	27	25	24	25	25
P. ramorum	MR-88	67	15	27	26	28	29	11	14
P. ramorum	MR-88	67	40	25	26	25	25	26	27
P. ramorum	MR-88	67	80	20	19	18	20	19	20
P. ramorum	MR-88	67	200	13	15	17	15	15	13
P. ramorum	MR-88	67	500	10	10	10	10	10	10
P. ramorum	MR-126	68	0	20	19	20	20	20	19
P. ramorum	MR-126	68	15	13	13	12	12	12	13
P. ramorum	MR-126	68	40	13	11.5	11	11	11	11
P. ramorum	MR-126	68	80	10	11	9	9	10	10
P. ramorum	MR-126	68	200	9	9	7	8	8	8.5
P. ramorum	MR-126	68	500	8	7	7	7	7	7
P. ramorum	MR-268	70	0	17	18	19	18	23	24
P. ramorum	MR-268	70	15	14	15	16	16	16	17
P. ramorum	MR-268	70	40	12	11	11	12	10	11
P. ramorum	MR-268	70	80	8	9	8	8	8	9
P. ramorum	MR-268	70	200	9	10	10	10	10	10
P. ramorum	MR-268	70	500	7	7	7	7	7	7

				Diameter (mm) including 5mm inoculum plug					
Species	Isolate name	Isolate number	Treatment (µg/mL phosphite)	rep 1		rep 2		rep 3	
P. ramorum	MR-270	71	0	17	16	17	18	13	13
P. ramorum	MR-270	71	15	17	17	18	19	18	19
P. ramorum	MR-270	71	40	17	18	19	17	18	20
P. ramorum	MR-270	71	80	10	9	9	10.5	10	10
P. ramorum	MR-270	71	200	9	9	10	9	10	9
P. ramorum	MR-270	71	500	7	8	7	8	7	7
P. ramorum	MR-187	72	0	28	28	27	27	25	25
P. ramorum	MR-187	72	15	10	13	11	13	15	13
P. ramorum	MR-187	72	40	10	12	10	10	14	11
P. ramorum	MR-187	72	80	11	14	13	12	13	12
P. ramorum	MR-187	72	200	10	10	9	10	10	10
P. ramorum	MR-187	72	500	7	7	7	7	7	7
P. ramorum	MR-196	73	0	15	15	17	15	17	18
P. ramorum	MR-196	73	15	21	22	20	19	18	18
P. ramorum	MR-196	73	40	21	18	18	21	17	19
P. ramorum	MR-196	73	80	8	9	8	9	8	9
P. ramorum	MR-196	73	200	9	9	9	9	10	9
P. ramorum	MR-196	73	500	7	7	7	10	7	7
P. ramorum	1461	74	0	18	19	16	17	19	18
P. ramorum	1461	74	15	20	20	18	19	18	19
P. ramorum	1461	74	40	19	16	19	17	20	18
P. ramorum	1461	74	80	10	9	10	9	9	8
P. ramorum	1461	74	200	9	9	7	9	9	9.5
P. ramorum	1461	74	500	7.5	7.5	8	7.5	9	7
P. syringae	KDA_RT9	75	0	38	38	39	40	40	40
P. syringae	KDA_RT9	75	15	24.5	23	21	20	20	20
P. syringae	KDA_RT9	75	40	17	18	17	18	17	18
P. syringae	KDA_RT9	75	80	17	17	17	17	16	16
P. syringae	KDA_RT9	75	200	18	18	17	17	17	18
P. syringae	KDA_RT9	75	500	13	13	11	12	11	11
P. syringae	SM15FEB_5CRP	76	0	26	26	24	24	24	24
P. syringae	SM15FEB_5CRP	76	15	12	12.5	12	12	12	14
P. syringae	SM15FEB_5CRP	76	40	14	13	14	13	13	14
P. syringae	SM15FEB_5CRP	76	80	9	10	8	9	8	9
P. syringae	SM15FEB_5CRP	76	200	9	9	8	8	8	8
P. syringae	SM15FEB_5CRP	76	500	7	7	6	7	6	6
P. syringae	SM15APR_B0V	77	0	26	26	23	25	28	27
P. syringae	SM15APR_B0V	77	15	17	15	15	15	15	15
P. syringae	SM15APR_B0V	77	40	16	16	16	14	15	14
P. syringae	SM15APR_B0V	77	80	15	15	13	14	13	14
P. syringae	SM15APR_B0V	77	200	14	15	14	15	15	16
P. syringae	SM15APR_B0V	77	500	11	11	10	10	11	11
P. syringae	SM15FEB_HOP	78	0	27	25	25	24	24	26
P. syringae	SM15FEB_HOP	78	15	13	13	13	12	12	12
P. syringae	SM15FEB_HOP	78	40	11.5	10	10	10	10	10
P. syringae	SM15FEB_HOP	78	80	14	13	11	14	12	11

				Diameter (mm) including 5mm inoculum plug					
**Species**	**Isolate name**	**Isolate number**	**Treatment (µg/mL phosphite)**	**rep 1**		**rep 2**		**rep 3**	
P. syringae	SM15FEB_HOP	78	200	12	13	9	10	11	12
P. syringae	SM15FEB_HOP	78	500	7	8	7	7	6	7
P. syringae	BSP2014_502	79	0	18	18	19	18	20	20
P. syringae	BSP2014_502	79	15	14	15	16	13	15	14
P. syringae	BSP2014_502	79	40	13	12	11	13	13	14
P. syringae	BSP2014_502	79	80	15	16	11	11	11	11
P. syringae	BSP2014_502	79	200	9	9	8	9	16	16
P. syringae	BSP2014_502	79	500	6	6	7	5	5.5	5.5
P. syringae	MP-12	59	0	34	33	33	33	34	33
P. syringae	MP-12	59	15	21	21	21	20	18	18
P. syringae	MP-12	59	40	18	16	15	17	14	16
P. syringae	MP-12	59	80	16	16	16	15	15	15
P. syringae	MP-12	59	200	15	13	19	18	15	13
P. syringae	MP-12	59	500	14	14	13	13	13	14
P. syringae	MP-15	60	0	26	26	24	25	25	26
P. syringae	MP-15	60	15	20	18	19	19	20	19
P. syringae	MP-15	60	40	13	15	13	14	16	14
P. syringae	MP-15	60	80	17	18	19	17	17	18
P. syringae	MP-15	60	200	13	14	13	14	16	15
P. syringae	MP-15	60	500	9	10	8	8	9	7
