# Supplementary material for: Evidence for rapid adaptive evolution of tolerance to chemical treatments in Phytophthora species and its practical implications
Source: PLoS One. 2018 Dec 10;13(12):e0208961. doi: 10.1371/journal.pone.0208961 (PMC6287812; doi:10.1371/journal.pone.0208961)
Supplement: S2 Table — Treatment is phosphite concentration (μg/mL). Blank square is no measurement. (PDF) [file pone.0208961.s002.pdf]

				Diameter (mm) including inoculum plug (5mm)					
Species	Isolate name	Isolate number	Treatment	rep 1		rep2		rep 3	
P. lateralis	PL-25	53	0	8	8	8	8	9	9
P. lateralis	PL-25	53	15	9	9.5	8	9	9	9
P. lateralis	PL-25	53	40	8.5	9	9	9	9	8
P. lateralis	PL-25	53	80	7.5	7	7	6	7.5	7
P. lateralis	PL-25	53	200	7	8	7	7	8	7
P. lateralis	PL-25	53	500	7	5	7	5	7	5
P. lateralis	PL-31	55	0	11	11	9	9	11	11
P. lateralis	PL-31	55	15	10	10	10	10	10	11
P. lateralis	PL-31	55	40	9	9	8	8	8	9
P. lateralis	PL-31	55	80	7	7.5	7	7	7	6.5
P. lateralis	PL-31	55	200	8	7.5	7	7	7	7
P. lateralis	PL-31	55	500	6	6.5	6	6	6	5.5
P. lateralis	PL-54	58	0	7	7	7	8	8	7
P. lateralis	PL-54	58	15	7	7	7	7	9	9
P. lateralis	PL-54	58	40	8	5.5	6	6	6	6
P. lateralis	PL-54	58	80	5.5	5	5	5.5	6	5
P. lateralis	PL-54	58	200	5	5	5	5	5	5
P. lateralis	PL-54	58	500	5	5	5	5	5	5
P. lateralis	PL-9	52	0	27	27	26	25	26	25
P. lateralis	PL-9	52	15			13	14	13	13.5
P. lateralis	PL-9	52	40	7	7	7	7	7	7
P. lateralis	PL-9	52	80	7	7	7	7	7	7
P. lateralis	PL-9	52	200	7	7	8	7.5	7	8
P. lateralis	PL-9	52	500	7	6	6	6	7	6.5
P. lateralis	Pl-28	54	0	25	25	24	24	25	25
P. lateralis	Pl-28	54	15	14	14	13	13	13	14
P. lateralis	Pl-28	54	40	7	7	6	6	6	6
P. lateralis	Pl-28	54	80	6	7	5.5	5.5	6	6
P. lateralis	Pl-28	54	200	6	6	5.5	5.5	5	6
P. lateralis	Pl-28	54	500	5	5	5	5	5.5	5.5
P. lateralis	PL-34	56	0	21	21	20	20	21	21
P. lateralis	PL-34	56	15	12	12	12	12	11	12
P. lateralis	PL-34	56	40	7	7	7	6	6.5	6.5
P. lateralis	PL-34	56	80	8	8	7	7	7	7
P. lateralis	PL-34	56	200	7	7	6	6	7	7
P. lateralis	PL-34	56	500	6	6	5.5	5	6	6
P. lateralis	PL-47	57	0	18	17	16	16		
P. lateralis	PL-47	57	15	10	10	10	9		
P. lateralis	PL-47	57	40	7	6	8	7		
P. lateralis	PL-47	57	80	9	7	7	7		
P. lateralis	PL-47	57	200	8	8	7	7		
P. lateralis	PL-47	57	500	6	6	7	7		
